# *N*-(2,6-Dimethyl­phen­yl)-4-methyl­benzamide

**DOI:** 10.1107/S1600536809022648

**Published:** 2009-06-17

**Authors:** B. Thimme Gowda, Miroslav Tokarčík, Jozef Kožíšek, B. P. Sowmya, Hartmut Fuess

**Affiliations:** a Department of Chemistry, Mangalore University, Mangalagangotri 574 199, Mangalore, India; b Faculty of Chemical and Food Technology, Slovak Technical University, Radlinského 9, SK-812 37 Bratislava, Slovak Republic; c Institute of Materials Science, Darmstadt University of Technology, Petersenstrasse 23, D-64287 Darmstadt, Germany

## Abstract

In the mol­ecular structure of the title compound, C_16_H_17_NO, the two aromatic rings are close to orthogonal to each other [dihedral angle 78.8 (1)°], while the central –NH—C(=O)– amide core is nearly coplanar with the benzoyl ring, forming a dihedral angle of 3.5 (2)°. Inter­molecular N—H⋯O hydrogen bonds in the crystal structure link the mol­ecules into infinite chains running along the *c* axis of the crystal, and a C—H⋯O interaction also occurs.

## Related literature

For  the preparation of the title compound, see: Gowda *et al.* (2003 ▶). For related structures, see: Gowda, Foro *et al.* (2008 ▶, 2009 ▶); Gowda, Tokarčík *et al.* (2008 ▶).
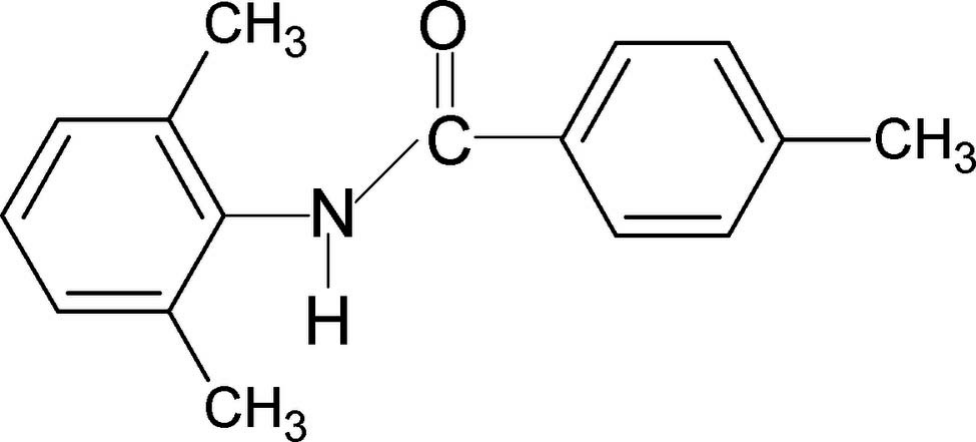


## Experimental

### 

#### Crystal data

C_16_H_17_NO
*M* *_r_* = 239.31
Tetragonal, 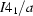

*a* = 16.6224 (5) Å
*c* = 19.9508 (7) Å
*V* = 5512.5 (3) Å^3^
*Z* = 16
Mo *K*α radiation
μ = 0.07 mm^−1^
*T* = 295 K
0.48 × 0.07 × 0.07 mm

#### Data collection

Oxford Diffraction Xcalibur diffractometer
Absorption correction: multi-scan (*CrysAlis RED*; Oxford Diffraction, 2008 ▶) *T*_min_ = 0.977, *T*_max_ = 0.992
17659 measured reflections
2649 independent reflections
1250 reflections with *I* > 2σ(*I*)
*R*_int_ = 0.039

#### Refinement

*R*[*F* ^2^ > 2σ(*F* ^2^)] = 0.038
*wR*(*F* ^2^) = 0.108
*S* = 0.99
2649 reflections
169 parameters
2 restraints
H atoms treated by a mixture of independent and constrained refinement
Δρ_max_ = 0.09 e Å^−3^
Δρ_min_ = −0.12 e Å^−3^

### 

Data collection: *CrysAlis CCD* (Oxford Diffraction, 2008 ▶); cell refinement: *CrysAlis RED* (Oxford Diffraction, 2008 ▶); data reduction: *CrysAlis RED*; program(s) used to solve structure: *SHELXS97* (Sheldrick, 2008 ▶); program(s) used to refine structure: *SHELXL97* (Sheldrick, 2008 ▶); molecular graphics: *ORTEP-3* (Farrugia, 1997 ▶) and *DIAMOND* (Brandenburg, 2002 ▶); software used to prepare material for publication: *SHELXL97*, *PLATON* (Spek, 2009 ▶) and *WinGX* (Farrugia, 1999 ▶).

## Supplementary Material

Crystal structure: contains datablocks I, global. DOI: 10.1107/S1600536809022648/dn2462sup1.cif

Structure factors: contains datablocks I. DOI: 10.1107/S1600536809022648/dn2462Isup2.hkl

Additional supplementary materials:  crystallographic information; 3D view; checkCIF report

## Figures and Tables

**Table 1:** Hydrogen-bond geometry (Å, °)

*D*—H⋯*A*	*D*—H	H⋯*A*	*D*⋯*A*	*D*—H⋯*A*
N1—H1N⋯O1^i^	0.892 (13)	2.025 (14)	2.8814 (16)	160.6 (15)
C7—H7⋯O1^i^	0.93	2.48	3.385 (2)	165

Symmetry code: (i) 
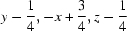
.

**Fig. 1.**
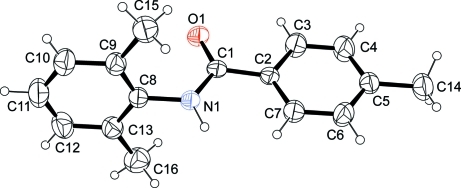
Molecular structure of (I) showing the atom labelling scheme. Displacement ellipsoids are drawn at the 30% probability level and H atoms are represented as small spheres of arbitrary radii.

**Fig. 2.**
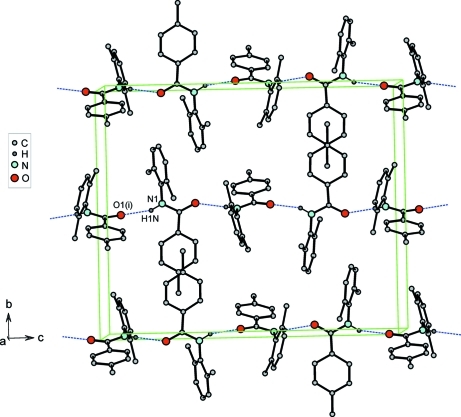
Part of the crystal structure of (I), showing the formation of hydrogen-bonded chains running in [001] direction. Symmetry code (i): y - 1/4, -x + 3/4, z - 1/4. H atoms not involved in hydrogen bonding have been omitted.

## supplementary crystallographic information

### Comment

As part of a study of the substituent effects on the crystal structures of benzanilides (Gowda, Foro *et al.*, 2008, 2009; Gowda, Tokarčík *et al.*, 2008), in the present work, the structure of 4-methyl-*N*-(2,6-dimethylphenyl)benzamide (I) has been determined. The conformations of the N—H and C═O bonds in the amide segment of the structure are anti to each other (Fig.1), similar to that observed in 4-methyl-*N*-(phenyl)benzamide (Gowda, Foro *et al.*, 2009), *N*-(2,6-dimethylphenyl)benzamide (Gowda, Tokarčík *et al.*, 2008), 2-methyl-*N*-(2,6-dimethylphenyl)benzamide (Gowda, Foro *et al.*, 2008) and other benzanilides, with similar bond parameters. The two aromatic rings in the structure of (I) make the dihedral angle of 78.8 (1)°, while the central amide core –NH—C(═O)– is nearly coplanar with the benzoyl ring, forming a dihedral angle of 3.5 (2)°. Part of the crystal structure of (I), showing the formation of hydrogen-bonded chains (Table 1) running in [001] direction is shown in Fig.2.

### Experimental

The title compound was prepared according to the method described by Gowda *et al.* (2003). The purity of the compound was checked by determining its melting point. It was characterized by recording its infrared and NMR spectra. Needle-like colourless single crystals of the title compound were obtained by slow evaporation from an ethanol solution of the compound (0.5 g in about 30 ml of ethanol) at room temperature.

### Refinement

All H atoms except amide H atom were placed in calculated positions with C—H distances in the range 0.93–0.96 Å and constrained to ride on their parent atoms. The C14 methyl group was refined as orientationally disordered using the instruction AFIX 127. Amide H atom was seen in difference map and was refined with the N—H distance restrained to 0.86 (2) Å. The *U*_iso_(H) values were set at 1.2*U*_eq_(C-aromatic,N) or 1.5*U*_eq_(C-methyl).

### Crystal data

**Table:**

C_16_H_17_NO	*D*_x_ = 1.153 Mg m^−^^3^
*M**_r_* = 239.31	Mo *K*α radiation, λ = 0.71073 Å
Tetragonal, *I*4_1_/*a*	Cell parameters from 3736 reflections
Hall symbol: -I 4ad	θ = 3.2–29.6°
*a* = 16.6224 (5) Å	µ = 0.07 mm^−^^1^
*c* = 19.9508 (7) Å	*T* = 295 K
*V* = 5512.5 (3) Å^3^	Needle, colourless
*Z* = 16	0.48 × 0.07 × 0.07 mm
*F*(000) = 2048	

### Data collection

**Table:**

Oxford Diffraction Xcalibur diffractometer	2649 independent reflections
graphite	1250 reflections with *I* > 2σ(*I*)
Detector resolution: 10.434 pixels mm^-1^	*R*_int_ = 0.039
ω scans with κ offsets	θ_max_ = 25.8°, θ_min_ = 3.2°
Absorption correction: multi-scan (*CrysAlis RED*; Oxford Diffraction, 2008)	*h* = −19→20
*T*_min_ = 0.977, *T*_max_ = 0.992	*k* = −18→20
17659 measured reflections	*l* = −24→24

### Refinement

**Table:**

Refinement on *F*^2^	Primary atom site location: structure-invariant direct methods
Least-squares matrix: full	Secondary atom site location: difference Fourier map
*R*[*F*^2^ > 2σ(*F*^2^)] = 0.038	Hydrogen site location: inferred from neighbouring sites
*wR*(*F*^2^) = 0.108	H atoms treated by a mixture of independent and constrained refinement
*S* = 0.99	*w* = [exp(2.10(sinθ/λ)^2^)]/[σ^2^(*F*_o_^2^) + (0.0579*P*)^2^], where *P* = 0.33333*F*_o_^2^ + 0.66667*F*_c_^2^
2649 reflections	(Δ/σ)_max_ < 0.001
169 parameters	Δρ_max_ = 0.09 e Å^−^^3^
2 restraints	Δρ_min_ = −0.12 e Å^−^^3^

### Special details

**Table:**

Geometry. All e.s.d.'s (except the e.s.d. in the dihedral angle between two l.s. planes) are estimated using the full covariance matrix. The cell e.s.d.'s are taken into account individually in the estimation of e.s.d.'s in distances, angles and torsion angles; correlations between e.s.d.'s in cell parameters are only used when they are defined by crystal symmetry. An approximate (isotropic) treatment of cell e.s.d.'s is used for estimating e.s.d.'s involving l.s. planes.
Refinement. Refinement of *F*^2^ against ALL reflections. The weighted *R*-factor *wR* and goodness of fit *S* are based on *F*^2^, conventional *R*-factors *R* are based on *F*, with *F* set to zero for negative *F*^2^. The threshold expression of *F*^2^ > σ(*F*^2^) is used only for calculating *R*-factors(gt) *etc*. and is not relevant to the choice of reflections for refinement. *R*-factors based on *F*^2^ are statistically about twice as large as those based on *F*, and *R*-factors based on ALL data will be even larger.

### Fractional atomic coordinates and isotropic or equivalent isotropic displacement parameters (Å^2^)

**Table:**

	*x*	*y*	*z*	*U*_iso_*/*U*_eq_	Occ. (<1)
C1	0.27487 (9)	0.49299 (9)	0.25025 (7)	0.0506 (4)	
O1	0.26537 (8)	0.52765 (7)	0.30418 (5)	0.0697 (4)	
N1	0.25165 (9)	0.52730 (8)	0.19248 (6)	0.0573 (4)	
H1N	0.2648 (10)	0.5037 (9)	0.1539 (7)	0.069*	
C2	0.31055 (9)	0.41089 (9)	0.24642 (6)	0.0483 (4)	
C3	0.33373 (14)	0.37365 (12)	0.30424 (8)	0.0900 (7)	
H3	0.3289	0.4009	0.3448	0.108*	
C4	0.36405 (15)	0.29691 (13)	0.30384 (9)	0.0959 (7)	
H4	0.3789	0.2735	0.3443	0.115*	
C5	0.37308 (10)	0.25407 (10)	0.24670 (9)	0.0606 (5)	
C6	0.34988 (13)	0.29146 (12)	0.18953 (9)	0.0828 (6)	
H6	0.3548	0.264	0.1491	0.099*	
C7	0.31947 (13)	0.36797 (11)	0.18884 (8)	0.0769 (6)	
H7	0.3046	0.3911	0.1482	0.092*	
C8	0.21647 (11)	0.60581 (10)	0.18922 (7)	0.0565 (4)	
C9	0.13575 (12)	0.61467 (10)	0.20521 (7)	0.0630 (5)	
C10	0.10337 (13)	0.69090 (13)	0.20139 (10)	0.0801 (6)	
H10	0.0496	0.6988	0.2125	0.096*	
C11	0.14898 (17)	0.75510 (13)	0.18149 (11)	0.0937 (7)	
H11	0.126	0.806	0.1793	0.112*	
C12	0.22797 (16)	0.74490 (12)	0.16481 (10)	0.0885 (7)	
H12	0.2579	0.7889	0.1505	0.106*	
C13	0.26422 (12)	0.66992 (11)	0.16895 (9)	0.0700 (5)	
C14	0.40544 (13)	0.16975 (11)	0.24629 (11)	0.0866 (6)	
H14A	0.3916	0.1441	0.2047	0.13*	0.5
H14B	0.3825	0.1401	0.2829	0.13*	0.5
H14C	0.4629	0.1711	0.251	0.13*	0.5
H14D	0.4331	0.1594	0.2877	0.13*	0.5
H14E	0.4422	0.1635	0.2095	0.13*	0.5
H14F	0.3618	0.1324	0.2414	0.13*	0.5
C15	0.08424 (12)	0.54478 (13)	0.22548 (10)	0.0842 (6)	
H15A	0.0822	0.5064	0.1895	0.126*	
H15B	0.0309	0.5634	0.2353	0.126*	
H15C	0.1067	0.5197	0.2646	0.126*	
C16	0.35125 (14)	0.65809 (14)	0.15300 (11)	0.0971 (7)	
H16A	0.3778	0.6338	0.1906	0.146*	
H16B	0.3756	0.7092	0.1435	0.146*	
H16C	0.3563	0.6237	0.1146	0.146*	

### Atomic displacement parameters (Å^2^)

**Table:**

	*U*^11^	*U*^22^	*U*^33^	*U*^12^	*U*^13^	*U*^23^
C1	0.0587 (10)	0.0565 (10)	0.0367 (8)	0.0018 (8)	0.0004 (7)	0.0000 (7)
O1	0.1009 (10)	0.0724 (8)	0.0358 (6)	0.0166 (7)	−0.0008 (5)	−0.0073 (5)
N1	0.0802 (10)	0.0571 (9)	0.0347 (6)	0.0149 (7)	−0.0026 (6)	−0.0030 (6)
C2	0.0532 (9)	0.0524 (10)	0.0391 (8)	0.0019 (8)	0.0000 (7)	0.0014 (7)
C3	0.144 (2)	0.0805 (15)	0.0451 (10)	0.0401 (14)	−0.0106 (11)	0.0004 (9)
C4	0.149 (2)	0.0801 (15)	0.0588 (12)	0.0407 (15)	−0.0138 (12)	0.0123 (10)
C5	0.0590 (11)	0.0547 (11)	0.0681 (11)	0.0028 (8)	0.0006 (8)	0.0053 (9)
C6	0.1222 (18)	0.0660 (14)	0.0603 (11)	0.0237 (12)	−0.0004 (11)	−0.0090 (9)
C7	0.1186 (17)	0.0670 (13)	0.0450 (9)	0.0233 (11)	−0.0040 (10)	0.0010 (8)
C8	0.0777 (13)	0.0525 (11)	0.0392 (8)	0.0119 (10)	−0.0078 (8)	−0.0030 (7)
C9	0.0758 (14)	0.0587 (12)	0.0546 (10)	0.0104 (10)	−0.0076 (8)	−0.0021 (8)
C10	0.0811 (14)	0.0717 (15)	0.0874 (13)	0.0192 (12)	−0.0078 (11)	−0.0007 (11)
C11	0.116 (2)	0.0634 (15)	0.1019 (16)	0.0252 (15)	−0.0078 (14)	0.0029 (11)
C12	0.116 (2)	0.0550 (13)	0.0946 (14)	−0.0017 (13)	0.0019 (13)	0.0062 (10)
C13	0.0870 (15)	0.0604 (13)	0.0625 (10)	0.0017 (11)	−0.0007 (9)	−0.0021 (9)
C14	0.0941 (16)	0.0622 (13)	0.1034 (15)	0.0131 (11)	−0.0005 (12)	0.0093 (11)
C15	0.0811 (15)	0.0787 (14)	0.0928 (14)	0.0011 (12)	0.0017 (11)	0.0046 (10)
C16	0.0926 (17)	0.0928 (16)	0.1059 (16)	−0.0052 (13)	0.0143 (13)	−0.0034 (12)

### Geometric parameters (Å, °)

**Table:**

C1—O1	1.2307 (16)	C10—C11	1.368 (3)
C1—N1	1.3427 (17)	C10—H10	0.93
C1—C2	1.490 (2)	C11—C12	1.365 (3)
N1—C8	1.431 (2)	C11—H11	0.93
N1—H1N	0.892 (13)	C12—C13	1.387 (3)
C2—C7	1.360 (2)	C12—H12	0.93
C2—C3	1.365 (2)	C13—C16	1.494 (3)
C3—C4	1.372 (3)	C14—H14A	0.96
C3—H3	0.93	C14—H14B	0.96
C4—C5	1.352 (2)	C14—H14C	0.96
C4—H4	0.93	C14—H14D	0.96
C5—C6	1.355 (2)	C14—H14E	0.96
C5—C14	1.501 (2)	C14—H14F	0.96
C6—C7	1.369 (3)	C15—H15A	0.96
C6—H6	0.93	C15—H15B	0.96
C7—H7	0.93	C15—H15C	0.96
C8—C9	1.387 (2)	C16—H16A	0.96
C8—C13	1.389 (2)	C16—H16B	0.96
C9—C10	1.379 (2)	C16—H16C	0.96
C9—C15	1.499 (3)		
			
O1—C1—N1	120.98 (15)	C13—C12—H12	119.5
O1—C1—C2	121.65 (13)	C12—C13—C8	117.29 (19)
N1—C1—C2	117.35 (13)	C12—C13—C16	121.75 (19)
C1—N1—C8	122.94 (12)	C8—C13—C16	120.95 (18)
C1—N1—H1N	119.0 (11)	C5—C14—H14A	109.5
C8—N1—H1N	117.5 (10)	C5—C14—H14B	109.5
C7—C2—C3	116.44 (15)	H14A—C14—H14B	109.5
C7—C2—C1	124.54 (14)	C5—C14—H14C	109.5
C3—C2—C1	118.98 (14)	H14A—C14—H14C	109.5
C2—C3—C4	121.38 (16)	H14B—C14—H14C	109.5
C2—C3—H3	119.3	C5—C14—H14D	109.5
C4—C3—H3	119.3	H14A—C14—H14D	141.1
C5—C4—C3	122.37 (17)	H14B—C14—H14D	56.3
C5—C4—H4	118.8	H14C—C14—H14D	56.3
C3—C4—H4	118.8	C5—C14—H14E	109.5
C4—C5—C6	115.88 (16)	H14A—C14—H14E	56.3
C4—C5—C14	122.41 (17)	H14B—C14—H14E	141.1
C6—C5—C14	121.70 (17)	H14C—C14—H14E	56.3
C5—C6—C7	122.67 (16)	H14D—C14—H14E	109.5
C5—C6—H6	118.7	C5—C14—H14F	109.5
C7—C6—H6	118.7	H14A—C14—H14F	56.3
C2—C7—C6	121.27 (15)	H14B—C14—H14F	56.3
C2—C7—H7	119.4	H14C—C14—H14F	141.1
C6—C7—H7	119.4	H14D—C14—H14F	109.5
C9—C8—C13	122.57 (16)	H14E—C14—H14F	109.5
C9—C8—N1	118.78 (16)	C9—C15—H15A	109.5
C13—C8—N1	118.63 (17)	C9—C15—H15B	109.5
C10—C9—C8	117.55 (18)	H15A—C15—H15B	109.5
C10—C9—C15	120.28 (19)	C9—C15—H15C	109.5
C8—C9—C15	122.17 (16)	H15A—C15—H15C	109.5
C11—C10—C9	121.1 (2)	H15B—C15—H15C	109.5
C11—C10—H10	119.4	C13—C16—H16A	109.5
C9—C10—H10	119.4	C13—C16—H16B	109.5
C12—C11—C10	120.47 (19)	H16A—C16—H16B	109.5
C12—C11—H11	119.8	C13—C16—H16C	109.5
C10—C11—H11	119.8	H16A—C16—H16C	109.5
C11—C12—C13	121.0 (2)	H16B—C16—H16C	109.5
C11—C12—H12	119.5		
			
O1—C1—N1—C8	1.4 (3)	C1—N1—C8—C9	−79.3 (2)
C2—C1—N1—C8	179.93 (15)	C1—N1—C8—C13	101.84 (18)
O1—C1—C2—C7	177.04 (17)	C13—C8—C9—C10	−1.0 (2)
N1—C1—C2—C7	−1.5 (3)	N1—C8—C9—C10	−179.76 (14)
O1—C1—C2—C3	−0.5 (3)	C13—C8—C9—C15	178.53 (16)
N1—C1—C2—C3	−179.03 (17)	N1—C8—C9—C15	−0.2 (2)
C7—C2—C3—C4	−0.2 (3)	C8—C9—C10—C11	1.0 (3)
C1—C2—C3—C4	177.5 (2)	C15—C9—C10—C11	−178.47 (18)
C2—C3—C4—C5	0.3 (4)	C9—C10—C11—C12	0.1 (3)
C3—C4—C5—C6	−0.3 (3)	C10—C11—C12—C13	−1.4 (3)
C3—C4—C5—C14	−179.3 (2)	C11—C12—C13—C8	1.4 (3)
C4—C5—C6—C7	0.3 (3)	C11—C12—C13—C16	−178.01 (19)
C14—C5—C6—C7	179.3 (2)	C9—C8—C13—C12	−0.2 (2)
C3—C2—C7—C6	0.2 (3)	N1—C8—C13—C12	178.56 (15)
C1—C2—C7—C6	−177.35 (18)	C9—C8—C13—C16	179.20 (15)
C5—C6—C7—C2	−0.3 (3)	N1—C8—C13—C16	−2.0 (2)

### Hydrogen-bond geometry (Å, °)

**Table:**

*D*—H···*A*	*D*—H	H···*A*	*D*···*A*	*D*—H···*A*
N1—H1N···O1^i^	0.89 (1)	2.03 (1)	2.8814 (16)	161 (2)
C7—H7···O1^i^	0.93	2.48	3.385 (2)	165

Symmetry codes: (i) *y*−1/4, −*x*+3/4, *z*−1/4.
